# Unsupervised Machine Learning Applied to Seismic Interpretation: Towards an Unsupervised Automated Interpretation Tool

**DOI:** 10.3390/s21196347

**Published:** 2021-09-23

**Authors:** Alimed Celecia, Karla Figueiredo, Carlos Rodriguez, Marley Vellasco, Edwin Maldonado, Marco Aurélio Silva, Anderson Rodrigues, Renata Nascimento, Carla Ourofino

**Affiliations:** 1 Electrical Engineering Department, PUC-Rio, Rio de Janeiro 22451-900, Brazil; acelecia@aluno.puc-rio.br (A.C.); edmaldonadot@hotmail.com (E.M.); andersonrds.rj@gmail.com (A.R.); 2 Department of Informatics and Computer Science, Institute of Mathematics and Statistics, State University of Rio de Janeiro (UERJ), Rio de Janeiro 20550-900, Brazil; 3 Tecgraf Institute, PUC-Rio, Rio de Janeiro 22451-900, Brazil; carlosrodriguez@tecgraf.puc-rio.br (C.R.); rlins@tecgraf.puc-rio.br (R.N.); ourofino@tecgraf.puc-rio.br (C.O.); 4 Department of Electronics and Telecommunications, State University of Rio de Janeiro (UERJ), Rio de Janeiro 20550-900, Brazil; marcoground@gmail.com

**Keywords:** unsupervised machine learning, seismic interpretation, image segmentation, well logs clustering

## Abstract

Seismic interpretation is a fundamental process for hydrocarbon exploration. This activity comprises identifying geological information through the processing and analysis of seismic data represented by different attributes. The interpretation process presents limitations related to its high data volume, own complexity, time consumption, and uncertainties incorporated by the experts’ work. Unsupervised machine learning models, by discovering underlying patterns in the data, can represent a novel approach to provide an accurate interpretation without any reference or label, eliminating the human bias. Therefore, in this work, we propose exploring multiple methodologies based on unsupervised learning algorithms to interpret seismic data. Specifically, two strategies considering classical clustering algorithms and image segmentation methods, combined with feature selection, were evaluated to select the best possible approach. Additionally, the resultant groups of the seismic data were associated with groups obtained from well logs of the same area, producing an interpretation with aggregated lithologic information. The resultant seismic groups correctly represented the main seismic facies and correlated adequately with the groups obtained from the well logs data.

## 1. Introduction

A key aspect of any hydrocarbon exploration activity is understanding the subsurface structure and its properties in order to determine the existence of hydrocarbon deposits such as petroleum or natural gas. Geophysical exploration can be defined as the search for commercial deposits of useful minerals, including hydrocarbons [1]. As a fundamental process in exploration, seismic interpretation comprises the identification of geological information through the processing and analysis of seismic data [2]. Interpretation can also be considered as Deriving a simple, plausible geologic model that is compatible with all observed data. The model is never unique and refining it involves a sequence of somewhat arbitrary choices [1]. These data, after they are acquired, are processed by transforming them into different attributes that can highlight some geologic structure, stratigraphic feature, or rock properties. 

Usually, the number of generated attributes is in the order of tens [3], producing a considerable amount of data that can be redundant or irrelevant for a given task. Moreover, individual seismic attributes may be related to some subsurface features, helping their identification. Examples are root mean square amplitude for delineating direct hydrocarbon indicators [4] or geometric attributes for fault/fracture detection [5]. As a result, applying a multi-attribute analysis is suggested, in order to improve the outcome of the interpretation process.

An interpreter is a specialist who analyzes this large volume of data to define the most likely subsurface model and geological evolution through seismic facies analysis. Interpretation is an iterative process in which the expert has to employ his/her experience, skills, and knowledge in the area of interest to select the best set of attributes that describes a geological layer [6]. Therefore, interpreters should deeply understand each aspect of the available seismic data, its acquisition and processing, as well as the technologies employed for interpretation assistance and attributes generation [7]. This knowledge is particularly important for avoiding errors or common pitfalls in seismic data interpretation.

The interpretation process is then limited by its complexity, time consumption, uncertainties added by the subjectivity of the expert analysis, the quality of his/her work, and the limitations of the software employed in workstation-based interpretations. Moreover, the process should adequately integrate geological, geophysical, petrophysical, and engineering data [2]. Such a huge volume of data to process can be challenging even for an experienced interpreter. Additionally, it can result in a suboptimal solution, producing losses in the order of millions of dollars during hydrocarbon’s exploration [8,9].

An alternative approach for this process is the application of machine learning algorithms on seismic data. Such techniques can contribute to speeding up the activity through an accurate interpretation that can serve as valuable assistance for the experts. For seismic signal classification tasks, many supervised machine learning models, such as Support Vector Machine (SVM) [10], Decision Trees [11], Multilayer Perceptron Neural Networks (MLP), or Convolutional Neural Networks [12,13], have been explored. These types of algorithms employ predefined labels (product of the human interpretation of seismic data) to train the models, optimizing their label recognition capacity in a given dataset. In this way, their success depends on the seismic data labels and, consequently, on the interpreter’s overall performance.

Unsupervised Machine Learning models, in contrast, are a category of algorithms that, without any reference or label, discovers underlying patterns and relationships in the data. Applying such a paradigm of algorithms can identify new relationships between seismic attributes, which are unknown beforehand, assisting the expert in generating a more accurate interpretation. A large amount of seismic data and the lack of labels available in new exploration [14] add to the advantages of applying these models. Moreover, the application of this type of algorithm can be extended to data other than seismic, such as well logs [15], obtaining the structures of lithological data. The association between those results and the seismic data can produce a more enriched and informative interpretation model.

This algorithm category can be separated into two main groups: classical clustering algorithms and image segmentation algorithms. Classical clustering algorithms have been applied successfully in the seismic and well data exploration areas separately. Examples are self-organizing maps [5], k-Means [16], Fuzzy c-Means [17], or Growing Neural Gas (GNG) [18], among others. Those studies concentrate on the proposal and evaluation of a single model, limiting their analysis by not validating or comparing their results with other state-of-the-art algorithms over the same data. A proper evaluation requires a comparison with other algorithms to contrast and discuss the results. Additionally, the advantage offered of applying a feature selection process is not commonly explored, and when included, is limited to classical models such as Principal Component Analysis (PCA) [5,19] or Independent Component Analysis (ICA) [16].

On the other hand, image segmentation algorithms are not as common as classical clustering algorithms in seismic interpretation problems. In general, their utilization is limited to segmenting a specific seismic object, as in [20,21] for salt boundaries or [22] for salt diapir. In these approaches, seismic slices are treated as image sequences that are processed independently. Therefore, a postprocessing step is required to combine the results in a seismic volume. More recently, Unsupervised Deep Learning architectures, such as Autoencoders or Generative Adversarial Networks (GANs), have been successfully applied on seismic interpretation problems [23,24,25], demonstrating similar or better performance than more traditional image segmentation methods. Additionally, feature dimensionality reduction [26] and multitask learning [27] have been recently proposed for high dimensional images processing.

One limitation of applying machine learning models to seismic data interpretation is the size of the data to be processed [28], which is usually in the order of tens of gigabytes (Gbs) [29]. When processed point to point, this amount of data makes it computationally impossible to apply such algorithms without a potent computing power [30]. Moreover, when complex Deep Learning architectures are utilized, the demanded computing power is even higher, requiring servers with multiples GPUs of proper memory to accommodate the Gbs of seismic data for more accurate and efficient 3D processing. A particular advantage of traditional image segmentation algorithms is the possibility to group data points considering spatial relationships, producing a novel representation that can alleviate the volume of the computing power demanded. Such groups can be associated through unsupervised algorithms or similarity functions to produce a unified result in the whole seismic volume. Given that it is only needed to process the groups, the computing power demanded is significantly reduced, without losing spatial information. A variant of such an approach was explored in [18], in which the GNG algorithm is employed to cluster amplitude voxels that are then associated using a similarity function.

In this work, we aim to explore the use of unsupervised machine learning models and image segmentation algorithms in a multi-attribute analysis for both seismic data and well logs. The main objective is to propose an automated seismic interpretation model to associate lithologic information obtained from groups with similar well log patterns to seismic facies. Both the seismic facies and the well logs groups are developed using unsupervised algorithms. 

To define the best model for seismic data, we evaluate two strategies for data representation: one directly clustering each point in the seismic volume (Pointwise Data Clustering); and another utilizing image segmentation algorithms to define spatial groups (Spatial Groups Data Clustering). Our proposal is based on the hypothesis that by employing unsupervised models, the geometrical and spatial/temporal relationships of seismic attributes (characterizing seismic facies patterns) can be correlated to lithologies defined by well log patterns. The obtained information can be applied to predict associated lithologic domains where no wellbore information is available in a fully automated procedure. Additionally, we assess several feature selection methods that can suggest the subset of seismic attributes more relevant for the description of the seismic data without redundancies in a pure algorithmic analysis. The development and evaluation of these models took into account the input of a specialist (geophysicist), who provided a valuable opinion throughout the work. Different configurations are evaluated and, based on the results obtained, the best approaches in each stage of the processing pipelines are defined in the final unsupervised interpretation model.

Therefore, the main contribution of this paper is the proposition of an interpretation model that can automatically discover the main seismic facies in the seismic data and associate them to lithologic information from well logs of the area. The interpretation model is based on unsupervised algorithms to process both seismic and well logs data. The proposed seismic data processing pipeline is described in detail, evaluating and comparing multiple models of unsupervised seismic interpretation that demand smaller computational power than Deep Learning models. Finally, the proposed interpretation model considers three unsupervised association methods between the groups formed in each domain (seismic and well logs data).

The remainder of this paper is organized as follows: Section 2 describes the materials and methods employed to develop the algorithms utilized in the data processing pipeline; Section 3 describes the results obtained from all the approaches used in the unsupervised interpretation model; finally, Section 4 provides the conclusions of the work.

## 2. Materials and Methods

The proposed unsupervised automated interpretation model is based on applying algorithms capable of recognizing patterns and relationships in the data without further information. The output of such a paradigm is the creation of different groups that separate the data that share an underlying pattern. The devised interpretation model explores the relationship between such clusters of seismic data and seismic logs to associate lithologic information to seismic facies. Therefore, the methodology is based on two independent data processing pipelines: one for the seismic data and the other for the well logs. The next subsection briefly introduces the theoretical aspects of seismic and well logs data, followed by subsections that describe each pipeline in detail.

### 2.1. Fundamentals of Seismic Reflection, Seismic Attributes and Well Logging

The main seismic method used in the oil industry is reflection. In this method, a source (explosives or vibrators on land, air-guns in marine environment) generates sonic waves, which propagate down into the Earth, are reflected at interfaces, propagate up, and are recorded by hundreds or thousands of receivers (geophones on land, hydrophones in marine) spread out on the surface.

For the reflection method, interfaces are boundaries (in general related to the top and base of geological layers) between two media with different levels of acoustic impedance (defined by the product of sound velocity and density). Velocity and density of rocks, layers, or sequence of layers, are defined by mineralogical content, and depositional and diagenetic (physical and chemical changes suffered by the rock after its deposition) process occurred in this rock along the geological time (from few thousands to hundreds of millions of years).

The amount of reflected energy at each interface is proportional to the acoustic impedance contrast between the layers above and below this interface. Thus, if acoustic impedance decreases from the upper to the lower layer, the amplitude (related to the square root of the reflected energy) is positive. This is common, as, in general, older rocks are below the newest ones, and these older rocks have, as a rule, higher velocities and densities. An interface of negative amplitude with high interest to hydrocarbon exploration is when the deeper layer is a rock with high porosity, and the pores are filled with oil or gas. Such a rock has relatively low velocity and density because the high number of pores makes wave propagation harder (decreasing velocity). The fluids filling the pores have a density much lower than the minerals of the rock.

In addition to the amplitude, key parameters that define the properties of upgoing reflected waves are travel time (how long it takes for a wave to travel from the source, be reflected, and be recorded by a receiver) and frequency content (as a wave travels through the Earth, its frequency content changes according to complex interaction among the wave front and the rocks it is propagating trough).

So, a final aspect of the seismic reflection data indicates some underground characteristics (e.g., kind of lithology, porosity amount, dominant fluid in rock pores, layer thickness, and age) fundamental for hydrocarbon exploration. As important as local characteristics, the vertical and lateral variations of these characteristics are key to indicate the presence of oil and/or gas in some area and the likelihood of a commercial accumulation. These characteristics are named seismic facies.

The final product of seismic acquisition and processing (necessary to correct wave propagation effects) is named as a section (for 2D) or cube (for 3D, to be used in this study). Both sections and cubes are made by hundreds or thousands of individual seismic traces grouped according to a fixed distance among them, with their geographical position being defined during seismic processing. All seismic traces have the same time length (varying between 4 to 10 s, corresponding to the time necessary for a reflected wave from the deepest interface of interest to arrive at the surface). The continuous reflected waves are discretized at a regular sampling interval (2 or 4 ms, so each seismic trace has 1000 to 5000 samples). Every sample has its correspondence to the amplitudes of the reflected waves.

From section or cubes, seismic attributes can be derived, aiming for better definition of layer limits and changes (both laterally and vertically). These attributes are extensively explained in the literature [2,6], derived from simple to complex mathematical operations applied in the “original” data. In general, seismic attributes may allow properties not clearly defined in the initial data to be identified, making them a useful tool for hydrocarbon exploration.

In this study, 13 attributes were used. Five of them (Frequency, Peak Frequency, Frequency 20 Hz, Frequency 30 Hz, Frequency 25/30/35 Hz) are related to frequency, four (Quadrature, Apparent Polarity, Instantaneous Phase, Cosine Phase) to complex trace, three (Amplitude, Peak Amplitude, Energy) to amplitude, and one (Chaos) to sharp lateral and/or vertical variations of seismic reflections and/or seismic facies, according to statistical analysis of reflection geometries.

Frequency indicates a characteristic of the amplitude spectrum (obtained by Fourier transform applied in the amplitude data), allowing the grouping of seismic facies with distinct spectra. Peak Frequency is, in the amplitude spectrum, the frequency value with the highest amplitude. Frequency 20 and 30 are, respectively, the resultant seismic data after a filter attenuates any frequencies below and above the desired one. Frequency 25/30/35 combines three datasets obtained after filtering the original data at 25, 30 and 35 Hz. This combination is sometimes called RGB, as it is common to associate frequencies with colors (Red, Green, and Blue) for display and analysis. It is also referred to as spectral decomposition, as the amplitude spectrum is decomposed in short frequency ranges. All frequency attributes are commonly used on seismic facies classification and clustering.

A complex trace is—as a complex number—a seismic trace with real and imaginary parts. The real trace is the original seismic trace obtained from seismic acquisition and processing, and the imaginary trace is the Hilbert transform of the real trace. Although the physical meaning of some complex attributes may be difficult to understand, their use is widespread as an additional source of information, being often useful as a “different view” (or approach) to a dataset. Quadrature is the imaginary trace, corresponding to a 90° phase shift (equivalent to the result of a Hilbert transform), with the same amplitude spectrum of real trace but sometimes highlighting subtle features. Apparent polarity is the sign (either positive or negative) for the complex trace maximum amplitude inside a time window. It can both individualize a thicker geological layer and indicate layer continuity when seismic data are noisy. The Instantaneous Phase is the phase of the complex trace at a specific time sample. As it does not depend on the amplitude, weak (low) and strong (high) amplitude reflections will be equally highlighted, providing interface continuity. This is often helpful in enhancing geometries of seismic reflections, which is a fundamental aspect of seismic facies. Cosine phase is the cosine of the Instantaneous Phase, with the same application but with the benefit of being continually smooth. 

Although seismic studies (including facies and attribute analysis) are routinely used in the industry as a necessary step for hydrocarbon exploration, a vital issue with using seismic data is that it is not a direct method. This issue produces a large amount of uncertainty on seismic analysis, as one has to use “second-hand” information on rock properties to estimate what is really important (volume of hydrocarbon in rock pores).

This issue is largely reduced when direct information is available. The main source of this direct information is drilled wells in the area where seismic data has been acquired and processed. The most important direct information comes from well logs, which measures the physical and chemical properties of geological layers drilled by the well. Common logs used in hydrocarbon exploration are Resistivity, Sonic, Shear, Density, Neutron, and Gamma-Ray.

As suggested by its name, Resistivity logs measure how much a rock opposes the electrical current inside it. Dominant fluid filling rock pores are saline (tens to few hundreds of ppm of salt) water, with a very low resistivity. Pores filled with gas or oil are much more resistive, so this log indicates hydrocarbon occurrence in a rock. Deep and medium values are related to how far the log measures resistivity from the wellbore. The difference between deep and medium values indicates how far the drilling mud (necessary for the well not to collapse during drilling) has penetrated the original rock.

Sonic and Shear logs measure, respectively, the slowness (inverse of velocity) of compressional (sound) and shear waves, so they are used to estimate physical properties (porosity and rock compaction are among the most important) that affects the velocity of these waves.

In the case of Density and Neutron logs, a source emits gamma rays close to the rock, dislodging electrons which are then deflected. Denser rocks have more electrons, generating more gamma rays scattering. A group of receivers measures the amount of gamma rays deflected by orbital electrons, indicating rock density, porosity and presence of gas.

Gamma Ray logs measure the natural radioactivity of the rocks. As a rule, the clay shales (very poor reservoirs) have natural radioactivity much higher than sandstones and carbonates. This occurs because clays are, in general, the most radioactivity minerals. Consequently, this log is commonly used to define if a rock is a good (very small shale or clay content) or bad (high shale or clay content) hydrocarbon reservoir.

Geologic layers have, in general, a constant pattern for Resistivity, Gamma Ray and Sonic/Shear, so these logs are used for layer correlation among wells. A quite extensive explanation of well logs in the hydrocarbon industry can be found in [31].

### 2.2. Seismic Data Analysis Methodology

**Figure 1:**
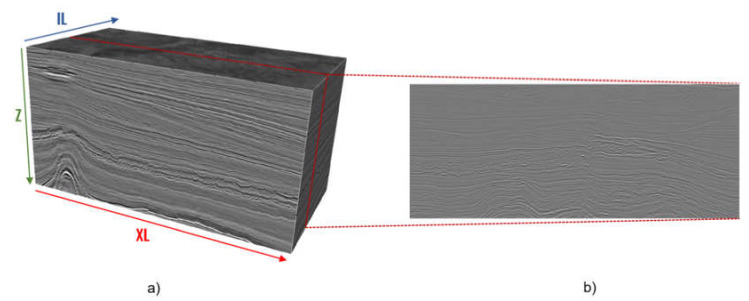
(**a**) Seismic Volume, (**b**) Slice (cut on the seismic volume) on a fixed *IL* coordinate.

The seismic data clustering models only require selecting a seismic volume of interest and calculating the seismic attributes to be included in the analysis. These data are represented by a seismic volume or cube with coordinates Inline (*IL*), Crossline (*XL*), and Domain (*Z*) [32], as can be seen in Figure 1a). A cut on the seismic volume in a fixed coordinate (*IL*, *XL*, or *Z*) is called a slice (Figure 1b).

**Figure 2:**
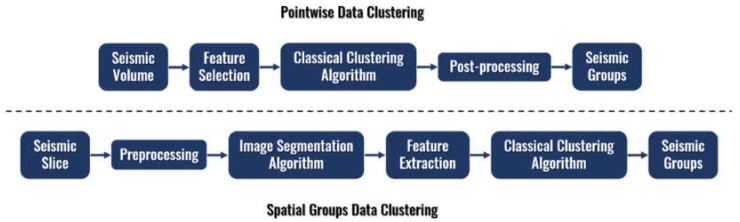
Proposed seismic data clustering approaches.

Given that a seismic volume can be considered a sequence of subsurface images, we propose two different approaches for seismic data clustering: Pointwise Data Clustering and Spatial Groups Data Clustering. The two alternatives are represented in Figure 2.

Pointwise Data Clustering analyzes the seismic volume using each data point $o\left(IL,\;XL,\;Z\right)$ as a sample. These samples can be described with multiple seismic attributes commonly extracted from the amplitude seismic data, such as energy, frequency, phase, among others. With the Seismic Volume usually requiring Gbs of storage, the addition of multiple seismic attributes for an accurate interpretation can escalate the volume of data to a prohibitive size for most Machine Learning algorithms. Therefore, we propose feature selection [33] to eliminate irrelevant or redundant seismic attributes, resulting in a significant reduction in the data size. Specifically, 28 seismic attributes are reduced to a subset of only 12 attributes employing the rankings provided by four feature selection algorithms: Principal Component Analysis [34], Principal Feature Analysis [35], Variance Threshold [36], and Feature weighting k-Means [37]. After selecting the best subset of seismic attributes, classical clustering algorithms are applied to the data, obtaining post-processed groups to enhance their homogeneity inside the seismic volume. These groups represent points in this multivariate seismic volume with similar seismic attributes values.

Spatial Groups Data Clustering, in contrast, defines a processing pipeline that employs image segmentation algorithms to define preliminary spatial groups of seismic data denominated segments. This approach begins by dividing the seismic volume in a sequence of slices in one of the coordinate directions. Each slice is represented as one image, which is then divided into multiple spatially compact segments through a segmentation algorithm. Then, several features are extracted from each segment, such as statistics of the seismic signals, texture, or shape information. Finally, a clustering algorithm is applied to the different segments based on the extracted features, separating them into groups with common characteristics. An in-depth description of the two methodologies is presented in the next subsections.

Therefore, both approaches presented in Figure 1b start the process from seismic attributes. However, the Pointwise Data Clustering approach creates seismic groups directly from the seismic attributes, while the Spatial Groups Data Clustering develops seismic groups from features extracted from segments created by an image segmentation algorithm using seismic attributes.

#### 2.2.1. Pointwise Data Clustering

The first stage of the pointwise data clustering approach is the feature selection process. Algorithms from feature selection models denominated filters [38] were selected to evaluate this strategy. This category includes the algorithms that rank the features or evaluate the subsets of features based on predefined parameters or metrics. The filter methods evaluated, which were selected considering their lightweight computing power demand, were: Principal Component Analysis (PCA), Principal Feature Analysis, Variance Threshold, and Feature weighting k-Means. In this approach, the features representing the seismic volume are directly the multiple seismic attributes.

The subsequent stage is the application of clustering algorithms to generate the groups of related seismic data from the seismic volume represented by the subset of seismic attributes. Our hypothesis is that these groups will be directly related to the seismic facies patterns of the data. The classical clustering models k-Means and Kohonen’s Self-Organizing Maps were evaluated in this work.

#### 2.2.2. Spatial Groups Data Clustering

**Figure 3:**
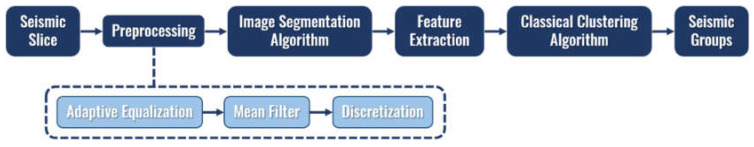
Image Preprocessing Pipeline.

**Figure 4:**
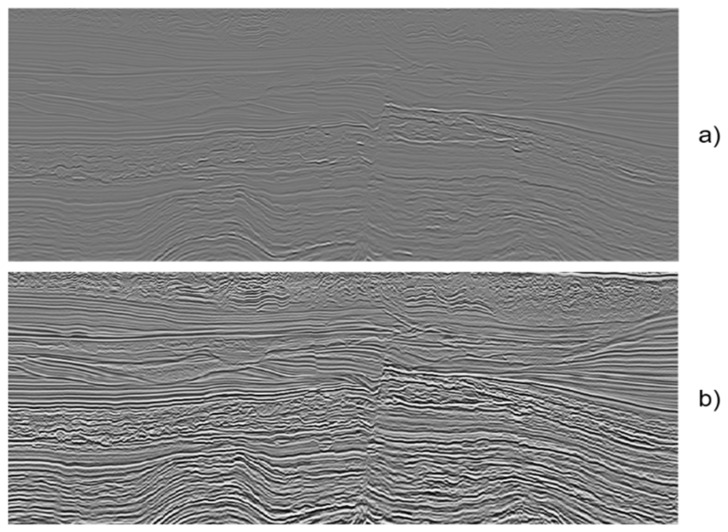
Example of results from the adaptive equalization on the seismic slice: (**a**) original seismic vertical slice, (**b**) seismic vertical slice after adaptive equalization.

This approach for seismic data clustering builds upon the seismic volume interpretation as a sequence of slices defining images of the seismic data in the inline, crossline, or domain coordinate. These seismic images can represent a single seismic attribute as a single channel grayscale image or multiple seismic attributes as the three channels of an RGB image. Given that the seismic data are a mixture of signals defined in the real number domain, it is necessary to apply a preprocessing stage to transform the seismic slice into a valid image. The diagram of the preprocessing steps is illustrated in the bottom part of Figure 3:

At first, the seismic slice distribution is enhanced using Adaptive Equalization [39], which improves the contrast of the image by transforming the intensity values, using the local information of the neighborhood, into an approximately uniform distribution. As a result, the details of the seismic data are highlighted, as can be seen in Figure 4. The equalization is followed by applying a Mean Filter, which eliminates noise that can be amplified during the Adaptive Equalization. After enhancing the contrast of the seismic data, the intensities are discretized in the range (0–255).

The next step in the Spatial Groups Data Clustering approach is the segmentation models, based on image segmentation algorithms, which aim to create spatially compact groups and similar colors denominated segments. Consequently, it is expected to generate segments that relate well to geologic structures observable in the seismic image. We evaluated two models with different principles for the image segmentation model: Watershed [40] and SLIC [41]. The former interprets the image as a three-dimensional topographical map, defined by the intensity values, that is gradually filled with water from seed points in the minimum points of the image. When water from different sources (basins) encounter, then a limit line is generated. The filling process ends when the water reaches the maximum point of the topography, producing segments associated with each basin. The latter is a superpixel algorithm that generates compact segments based on clustering intensities (employing k-Means) in a region proportional to the desired size of the superpixel. The distance function used in the k-Means algorithms is a combination of color and spatial proximity between the intensity values.

**Figure 5:**
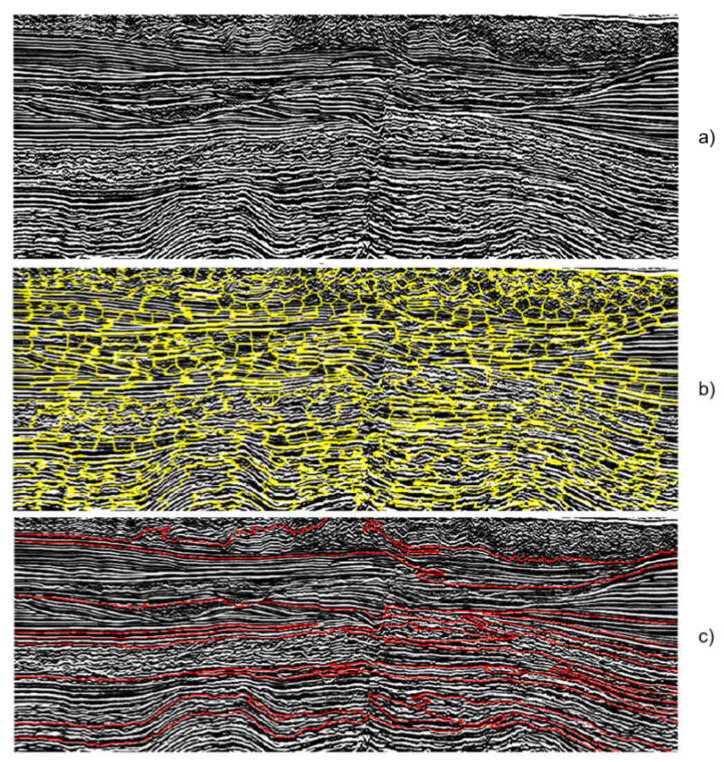
Example of results from the segmentation of the seismic (vertical slice) data (**a**) through the SLIC (**b**) and Watershed (**c**) algorithms.

To determine the best configuration for both SLIC and Watershed, we evaluated them with different numbers of segments and markers, respectively, in the range 15–1000. Figure 5 illustrates examples of segments obtained from both segmentation approaches. For SLIC, the seismic image is highly over-segmented, with compact segments that cannot always adhere to some of the main lithological features present in the image. On the other hand, the Watershed algorithm produces broader segments with better adherence to seismic facies patterns. In both approaches, it is visually noticeable inaccuracies between segments’ borders and facies which can be reduced in some level with the posterior clustering stage.

The resultant segments are spatially compact elements containing pixels with similarities between them. To cluster the obtained segments into regions of the image with common characteristics, relevant information from each segment must be extracted (Feature Extraction module in Figure 3). A clustering algorithm then uses the extracted features to determine the final groups (Classical Clustering Algorithm module in Figure 3).

**Figure 6:**
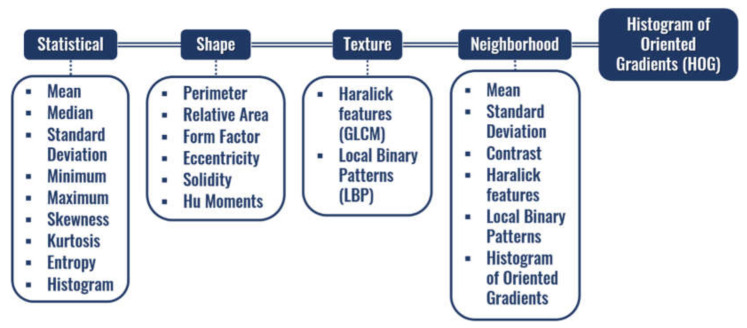
Set of features representing each segment of the Spatial Groups Data Clustering method.

Each segment is described through a combination of characteristics based on statistics of the seismic signals [42,43,44], shape [45,46,47,48], texture including Haralick features (GLCM) and Local Binary Patterns (LBP) [49,50,51], histogram of oriented gradients (HOG) [52,53], and Neighborhood information. These characteristics are represented in Figure 6 and are used in the clustering process employing any clustering algorithm. In this work, given that the volume of data to be clustered is reduced (the segments encompass several data points), we evaluated the following clustering algorithms: Agglomerative [54], Kohonen [55], and k-Means [56]. The result of these algorithms are groups with those image regions that are similar in the segments’ feature space and that, theoretically, should correlate with the seismic facies pattern in the images.

### 2.3. Well Logs Data Analysis Methodology

The seismic data employed in this work contain one well with sufficient size to be considered for the association process between lithological properties of the rocks and the seismic groups obtained through seismic data clustering. The lithological properties available in the well logs were: Deep Resistivity, Medium Resistivity, Sonic, Shear, Density, Neutron, and Gamma Ray. These logs, at first, are preprocessed following a windowing scheme in which all logs are divided into windows of a determined depth. Hence, each value at a depth x from the logs is represented by a window centered at x containing a neighborhood y around x. This strategy aggregates useful information for the clustering process in comparison to utilizing single values only. The windowed well log data are then clustered employing k-Means to find groups of similar lithologies.

### 2.4. Seismic and Well Logs Groups Association

The process for associating seismic facies, represented by the groups obtained from clustering the seismic data, with lithological properties represented through groups of well logs, follows these four steps:

i. Depth–time conversion to transform both datasets to the same domain (usually seismic data are given in the time domain and well logs in the depth domain);
ii. Blocking process to reduce the resolution of well logs data to the seismic data (well logs and seismic data present a resolution of 0.15 ms and 4 ms, respectively);
iii. Elimination of noisy segments resultant from previous operations (groups with a size smaller than 12 ms);
iv. Association between seismic and well logs groups.

The depth–time conversion employs the well velocity data and the depth (*Z*) to compute the two-way traveltime (*TWT*) information of each point using the expression:

(1) TWT=2*Z/Velocity

The blocking process is similar to the downsampling of a signal, decreasing the sample rate of well logs groups in the depth domain. This transformation is performed over the results of the clustering process of well logs data. The process divides these groups’ data into consecutive windows of 4 ms, which are represented with the label of the predominant group. After the downsampling process, to reduce spurious groups’ influence on the association results, sets with a size smaller than 12 ms (value defined by the geophysicist as adequate for this case) are considered noisy segments and, consequently, eliminated. Each eliminated depth interval then assumes the group label of its neighbor with the biggest size.

**Figure 7:**
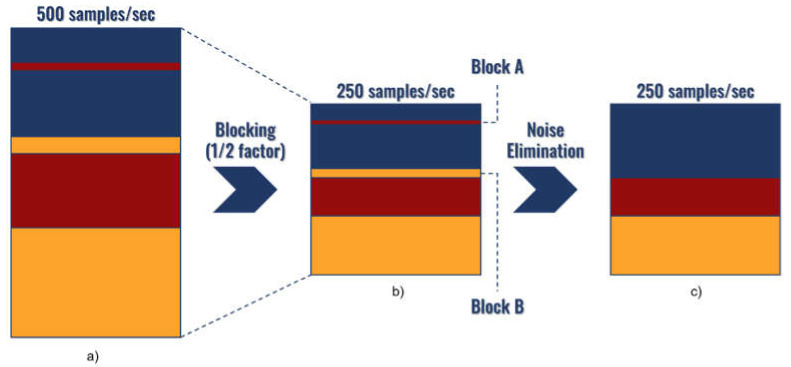
Pre-association steps: (**a**) original clustering considering samples of 2 ms, (**b**) result of the blocking process with a factor of 1/2 (each element of the column represents 4 ms on the original domain), (**c**) result of the noise elimination (eliminates the smaller red and orange sets that presented size of 4 and 8 ms, respectively). Above the columns, the correspodent sampling frequency is shown.

Figure 7 illustrates the set of pre-association steps in a hypothetical case. Figure 7a shows the well logs clustering result after converted to the time domain (considering samples of 2 ms). Figure 7b represents the result of the blocking process, and each element of the column represents 4 ms of the elements of the original domain. As a result, the resolution of the well logs groups is reduced in half. This process is followed by the inspection and elimination of noisy segments. Assuming the size of the smaller red and orange sets (Blocks A and B in Figure 7b) as 4 and 8 ms, respectively, which are smaller than the threshold (12 ms), these segments are considered noisy. Then, their labels are substituted by the one of their biggest neighbors (blue group). Finally, the resultant well logs clustering is shown in Figure 7c.

The obtained groups generalize the overall distribution of lithologies present in the well location in the same domain of the seismic data. Therefore, these groups can be associated with seismic groups by comparing their location in the subsurface. In this direction, we evaluated three different approaches: two based on the similarity between sample sets (Jaccard [57] and Rand Index [58]) and another based on a heuristic that employs the limits of each set.

The Jaccard Index and Rand Index are similarity measures between two sets commonly used in machine learning applications. The Jaccard Index measures the similarity between two groups by analyzing the relationship between their intersection over their union. Specifically, given:

- $f_{00}$: Number of pairs belonging to different well logs groups and seismic groups.
- $f_{01}$: Number of pairs belonging to different well logs groups and the same seismic groups.
- $f_{10}$: Number of pairs belonging to the same well logs groups and different seismic groups.
- $f_{11}$: Number of pairs belonging to the same well logs groups and seismic groups.

The Jaccard Index is defined as:

(2) $$JI=\frac{f_{11}}{f_{00}+f_{01}+f_{10}}$$

The Rand Index also computes the similarity between sets following the expression:

(3) $$RI=\frac{f_{00}+f_{11}}{f_{00}+f_{01}+f_{10}+f_{11}}$$

**Figure 8:**
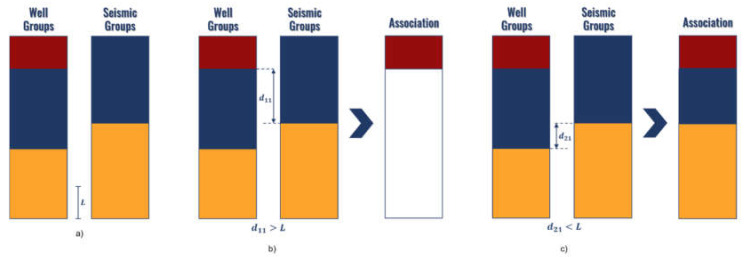
Exemplification of the association process evaluated from the top to the bottom of the well: (**a**) original clustering results, (**b**) association between the first limit of the well group and seismic group when the distance between group limits is bigger than a threshold *L*, (**c**) association between the second limit, which is now smaller than *L*.

The proposed heuristic to associate the obtained groups analyzes the limits of the different segments in the depth domain, as depicted in Figure 8. The process establishes an iterative search between the limits from well and seismic groups, starting from top to the bottom. At first, the first segment limit from the top of well group is located. When this limit is found, its distance to all partitions on the seismic groups is computed. The smallest one is compared to a defined threshold L (given in ms and represented in Figure 8a at the bottom to illustrate its size). This threshold is defined by the user depending on the resolution of the available data. If the distance surpasses L (meaning that there is no equivalent seismic group close enough in the seismic data results), then this set establishes a limit in the association results (Figure 8b). Conversely, if the distance is smaller than L, the limit from the set in the seismic side is assumed as associated with it and passes into the association results (Figure 8c). When all limits in the seismic groups are exhausted, the process ends and any group left in the well results passes its limits to the final association. The groups present in the association represent the approximated relationship between the spatial distribution of seismic and well groups.

In the figure, it can be observed that when there is no equivalent seismic group inside the distance threshold, the well group imposes its limit in the association result. This strategy is justified by the nature of the well log data, which are obtained directly from the subsurface and can be considered more reliable than the seismic data. Therefore, we considered maintaining well groups if an equivalent group is not present (the seismic data only produced a group in that region).

## 3. Results and Discussion

This section presents the case study used to evaluate the proposed unsupervised automated interpretation model comprising the two strategies for seismic data clustering (pointwise and spatial groups’ data clustering) and the results obtained from each processing stage. Given that the proposed models handle, in all stages, unlabeled data, the quality of the results obtained was evaluated by a specialist (geophysicist) for both seismic and well logs data. The seismic data used in this case study correspond to a seismic volume with dimensions: inline from 995 to 1775, crossline from 5613 to 7231, and domain 2200 to 3700 ms. Inline and crossline intervals are constant and equal to 25 and 12.5 m, so the area analyzed comprises a rectangle close to 400 km^2^ (19.5 km × 20.2 km). Examples of the groups obtained from the seismic data clustering are illustrated for the inline 1385 or 1413 (inlines in a close distance to the location of the well). All models were run in a Linux Server with CPU i7-5960X @ 3.00 GHz and 128 Gb of RAM memory.

### 3.1. Seismic Data Analysis

#### 3.1.1. Pointwise Data Clustering

**Figure 9:**
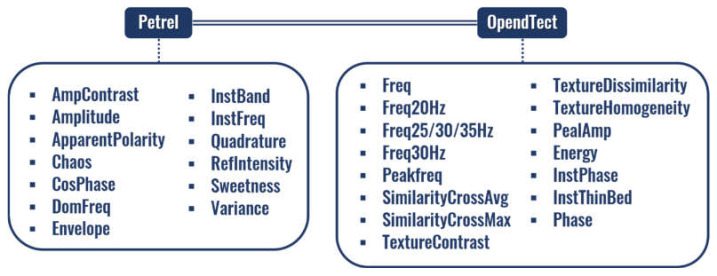
Seismic attributes.

**Figure 10:**
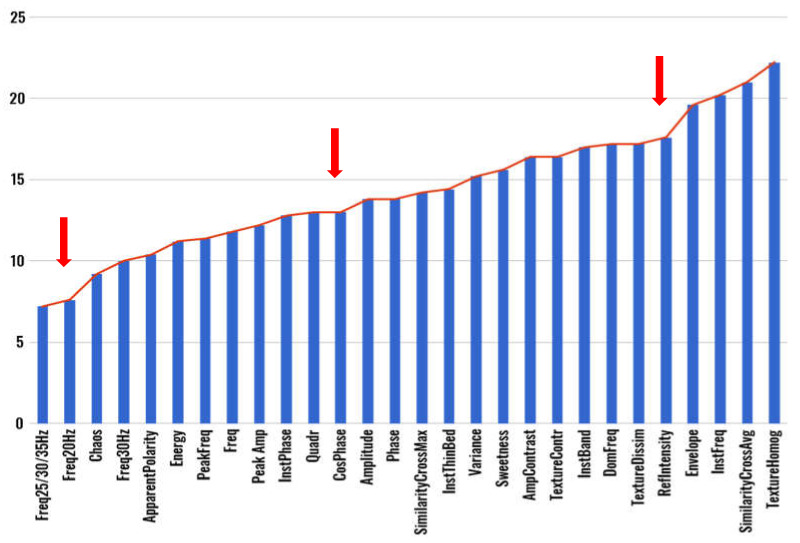
Mean rank of seismic attributes. Red arrows signal the most significant breaks.

The seismic volume is represented using a set of 28 seismic attributes computed using Petrel (https://www.software.slb.com/products/petrel, accessed on 22 March 2021) and OpendTect (https://www.dgbes.com/index.php/software/opendtect, accessed on 22 March 2021) software (Figure 9), which is then reduced by the feature selection stage. To select the best subset of seismic attributes, a ranking based on the results of the four feature selection algorithms mentioned in Section 2.1 (Principal Component Analysis, Principal Feature Analysis, Variance Threshold, and Feature weighting k-Means) is calculated. The mean of the ranking value of each seismic attribute obtained from each feature selection method is computed and is assumed as the importance of the attributes. The selected subset of attributes is determined by applying an approximation of the elbow method strategy [59]—originally employed to determine the number of clusters in an unsupervised learning analysis—to the mean ranking graph (observed in Figure 10). The most pronounced brakes are situated at the second (Freq20 Hz) and 24th (RefIntensity) attributes (first and third arrows in Figure 10). Nevertheless, these sets can be considered detrimental for the seismic analysis because only two frequency attributes do not identify all possible seismic structures and twenty-four does not significantly reduce the total number of attributes. Consequently, the next most significant break (the one with a marked disparity on the line inclination) was chosen, occurring at the 12th attribute (CosPhase). The selected subset then comprises those 12 attributes.

**Figure 11:**
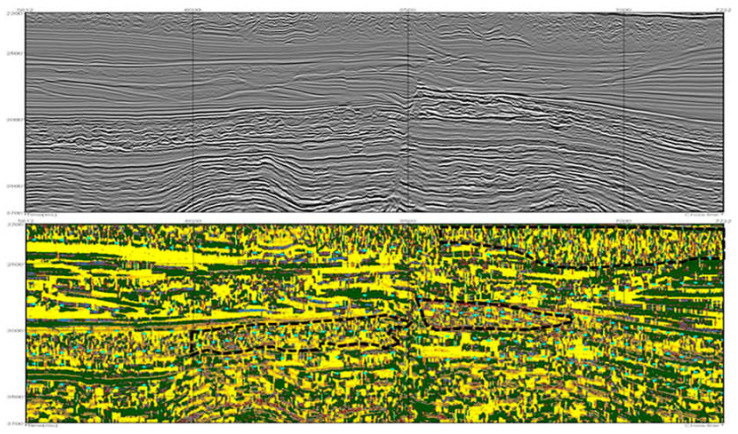
Results of the application of Kohonen on the best subset of seismic attributes with a rectangular map of size 6 × 6 neurons and 8 groups. At the top, the original vertical seismic slice is presented; on the bottom, each group from the Kohonen map is represented with a unique color. Black polygons indicate regions in which groups show a chaotic distribution.

**Figure 12:**
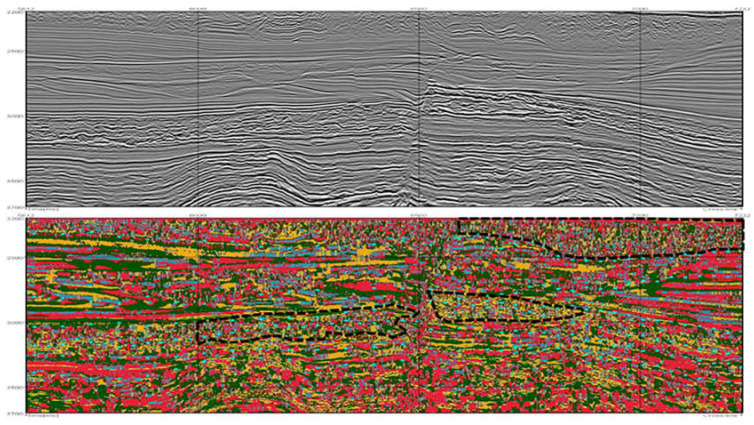
Results of the application of Kohonen on the best subset of seismic attributes and the Amplitude attribute with a rectangular map of size 6 × 6 neurons and 8 groups. The original vertical seismic slice is presented at the top; on the bottom, each group from the Kohonen map is represented with a unique color. Black polygons indicate regions in which groups show a chaotic distribution.

This subset of attributes is employed to cluster the seismic data in the pointwise clustering approach. From the unsupervised algorithms evaluated (k-Means and Kohonen), Kohonen resulted in more promising results, following the specialist’s assessment. Examples of one inline from the best results obtained are shown in Figure 11 and Figure 12, where each color indicates a different group from the clustering model. Figure 11 illustrates the resultant seismic groups from the application of Kohonen with eight groups. Following the specialist’s recommendation, adding the Amplitude attribute to the subset of features generated the result observed in Figure 12. The results did not significatively change, but they offered a slightly better definition of cluster separation, mainly in the deeper part of the figure (bottom part).

The groups obtained from the pointwise data clustering, as expected, delineate seismic components with common characteristics (these common characteristics are the seismic facies, explained in Section 2.1). Specifically, it is possible to differentiate diverse combinations of groups for the main seismic regions in the data. Nevertheless, in regions of the seismic data that present marked variability (examples are the superior and middle parts of Figure 11 and Figure 12 located inside the black polygons), the behavior of groups can be chaotic, presumably under the influence of the frequency attributes. Such a characteristic is not desirable for a seismic interpretation. Therefore, the performance of the pointwise clustering in combination with the best subset from seismic attributes is considered limited.

#### 3.1.2. Spatial Groups Data Clustering

The seismic data clustering based on spatial groups employs the segments from image segmentation algorithms (SLIC and Watershed) to obtain groups describing close distributions in space and magnitude from each seismic slice. Then, the model with the best results will be selected as part of the seismic data processing pipeline. As mentioned in Section 2.2.2 and observed in Figure 5, each algorithm produces segments with different characteristics. SLIC, on the one hand, over-segments the seismic data image and its segments do not adhere well to the main lithological features. On the other hand, the segments obtained by the Watershed algorithm are less compact, covering more regions of the seismic image with better accuracy concerning lithological features. The results of both segmentation algorithms were analyzed by the specialist (geophysicist), evaluating the quality of the obtained segments. The segments obtained employing Watershed present a better accuracy concerning seismic structures, covering a bigger area. Therefore, this algorithm was considered better for the proposed approach. It is important to highlight that the quality of the segments directly influences the result of the clustering algorithm, which enforces the criteria for selecting Watershed as the best method.

As described in Section 2.2.2, the segments obtained using the Watershed model on the seismic images are used in the clustering process. To represent each segment, several features were defined (illustrated in Figure 6). Each clustering algorithm considered (Agglomerative, Kohonen, and k-Means) was applied to the segments represented by a set composed of all these features. The parameters assessed for each clustering algorithm are summarized in Table 1.

The first set of experiments involved evaluating the best combination of three seismic attributes that provide the most informative seismic image, emulating the RGB color space. The evaluated combinations of seismic attributes are the following:

Amplitude, Quadrature, Sweetness.
Amplitude, Instantaneous Thin Bed, Sweetness.
Instantaneous Thin Bed, Similarity Cross Average, Energy.
CosPhase, Similarity Cross Average, Energy.
CosPhase, Amplitude, Energy.
Sweetness, Amplitude, Energy.
Amplitude, Apparent Polarity, Sweetness.
Amplitude, Amplitude Contrast, Similarity Cross Average.
Texture Dissimilarity, Amplitude, Similarity Cross Average.
Texture Contrast, Amplitude, Similarity Cross Average.
Texture Dissimilarity, Amplitude, Sweetness.
Amplitude, Texture Dissimilarity, Freq30 Hz.

These combinations were selected from tests exploring which attributes generated interesting results (several other combinations were unsuccessfully evaluated). From these combinations, the most interesting results were from the combinations {Texture Dissimilarity, Amplitude, Similarity Cross Average} and {Texture Contrast, Amplitude, Similarity Cross Average}.

**Figure 13:**
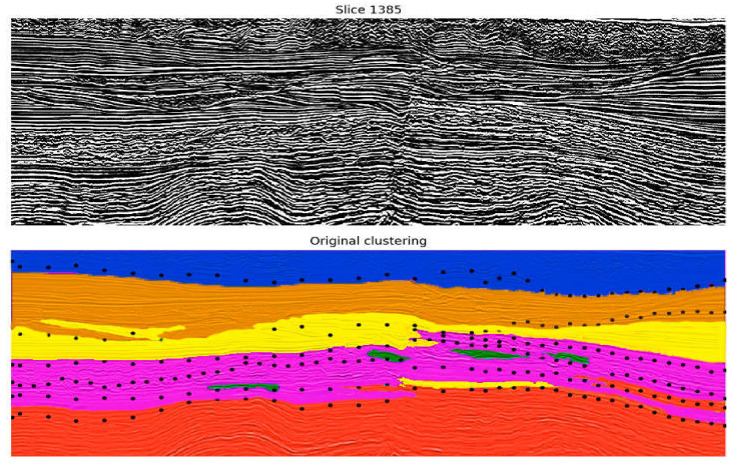
Results from the clustering of Watershed segments using k-Means (6 groups) and an image composed of Texture Dissimilarity, Amplitude, Similarity Cross Average. The top image shows the original vertical seismic slice; the bottom one shows the Watershed results. Black dots in the lower figure indicate main geological boundaries according to geophysicist’s interpretation of the top figure (conventional seismic data). One can conclude that a good fit occurs between clustering results and geophysicist’s preliminary interpretation.

**Figure 14:**
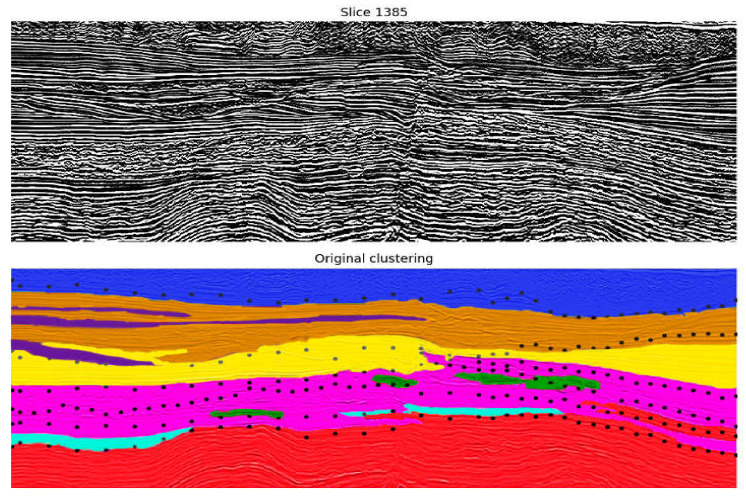
Results from the clustering of Watershed segments using k-Means (8 groups) and an image composed of Texture Dissimilarity, Amplitude, Similarity Cross Average. The top image shows the original vertical seismic slice; the bottom one shows the Watershed results. Black dots in the lower figure indicate main geological boundaries according to geophysicist interpretation of the top figure (conventional seismic data). One can conclude that a good fit occurs between clustering results and geophysicist’s preliminary interpretation.

**Figure 15:**
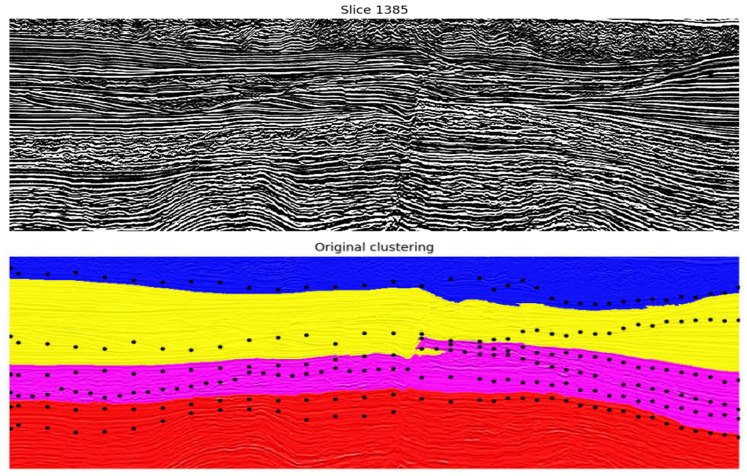
Results from the clustering of Watershed segments using k-Means (4 groups) and an image composed of Texture Contrast, Amplitude, Similarity Cross Average. The top image shows the original vertical seismic slice; the bottom one shows the Watershed results. Black dots in the lower figure indicate main geological boundaries according to geophysicist interpretation of the top figure (conventional seismic data). One can conclude that a good fit occurs between clustering results and geophysicist’s preliminary interpretation.

The selected clustering algorithms (Agglomerative, Kohonen, and k-Means) were applied to the obtained segments based on the two selected combinations. In all cases, the difference between clustering from the three algorithms was marginal. Therefore, the k-Means was selected as the most suitable algorithm, as it is the most computational efficient algorithm (shorter training time). Figure 13 and Figure 14 display two of the best results for the first combination and Figure 15 shows one for the second one.

The black points in each figure approximate the real limits between seismic facies, provided by the geophysics specialist. In general, it can be observed that in all examples, the groups obtained—defined with different colors in the image—encompass the main seismic facies of the data with acceptable accuracy.

A second analysis was performed to evaluate the best subset of features to represent the segments. This analysis aims to reduce the training time of the model, maintaining or improving the accuracy. The experiments to select the best subset of features followed the Forward Selection [60] approach based on the specialist’s opinion. Specifically, in each step, individual feature categories are employed in the clustering process. Based on the specialist´s evaluation of the results, the best category is included in the segment feature subset. The selection process is represented in Table 2, highlighting in each column the selected best category to be included in the feature subset. Based on the specialist’s suggestion, some single features from the overall categories that showed promising results independently were evaluated in some steps.

**Figure 16:**
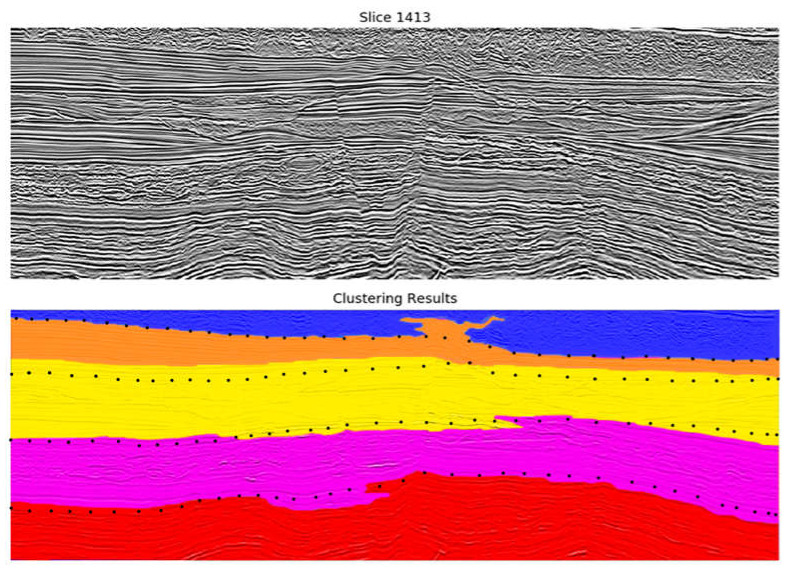
Results from the clustering of Watershed segments using k-Means (5 groups) with the best subset of features. The top image shows the original vertical seismic slice; the bottom one shows the Watershed results. Comparing this result with Figure 13, Figure 14 and Figure 15, it can be concluded that the accuracy of the model was improved.

**Figure 17:**
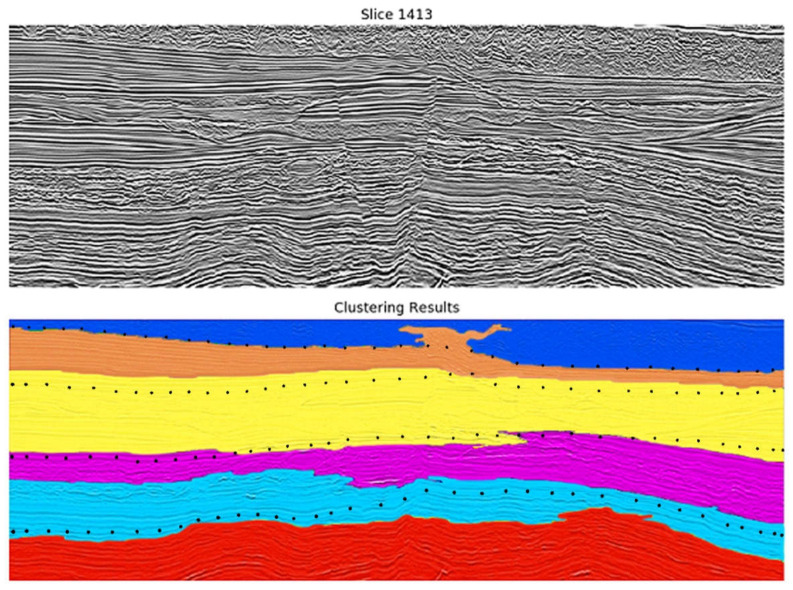
Results from the clustering of Watershed segments using k-Means (6 groups) with the best subset of features. The top image shows the original vertical seismic slice; the bottom one shows the Watershed results. Comparing this result with Figure 13, Figure 14 and Figure 15, it can be concluded that the accuracy of the model was improved.

Interestingly, the attributes selected in each step are somehow related to some type of texture description (HOG and LBP). Additionally, the information regarding the segments’ neighborhood is also meaningful for the clustering process. The clustering algorithms also produced similar results in this case, leading us to consider k-Means as the best algorithm due to its faster training time. The two most promising combinations of seismic attributes from the previous experiments were evaluated in this case. The one that produced better results was Texture Contrast, Amplitude, Similarity Cross Average. Examples of results from applying k-Means to the seismic data represented using the subset of segment features (HOG, Neighborhood HOG, LBP, Neighborhood LBP) are depicted in Figure 16 and Figure 17 (inline 1413).

As shown in Figure 16 and Figure 17, the subset of features was able to produce more homogeneous groups with good accuracy concerning the seismic structures. In contrast to the previous approach, smaller groups—which represent inaccuracies—were eliminated, which increments the final accuracy of the model. Nevertheless, the resultant groups are also limited when detailing some lithological structures that present similar characteristics, mainly present in the middle of the seismic image in the area inside the orange ellipse.

Compared to the pointwise clustering approach, this methodology presented a better accuracy concerning the main facies in the data, summarizing the data in broader and more homogeneous groups. Additionally, the training time of the learning models is significantly reduced, given that the segments encapsulate a significant number of data points.

### 3.2. Well Logs Data Analysis

**Figure 18:**
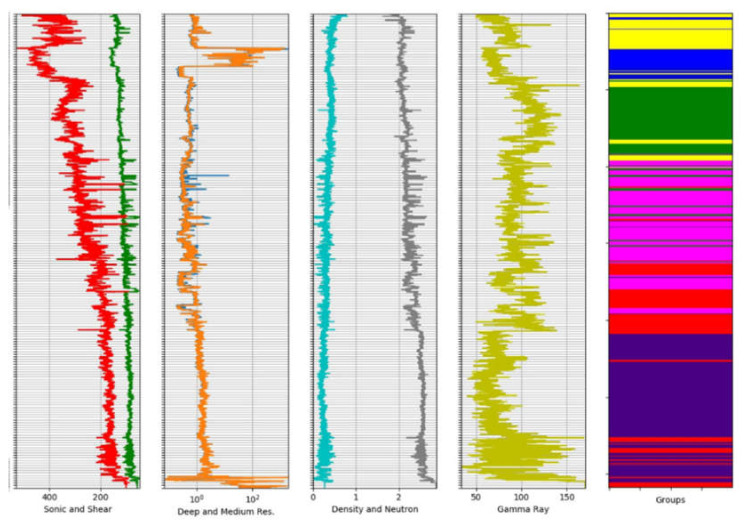
Groups obtained using k-Means (rightmost column) for 6 groups and a window of 6 m for well logs (from left to right, Sonic/Shear, Deep/Medium Resistivity, Density/Neutron and Gamma Ray). Clearly, the main lithologic groups (indicated by changes in well log patterns and values) were well defined by k-Means.

**Figure 19:**
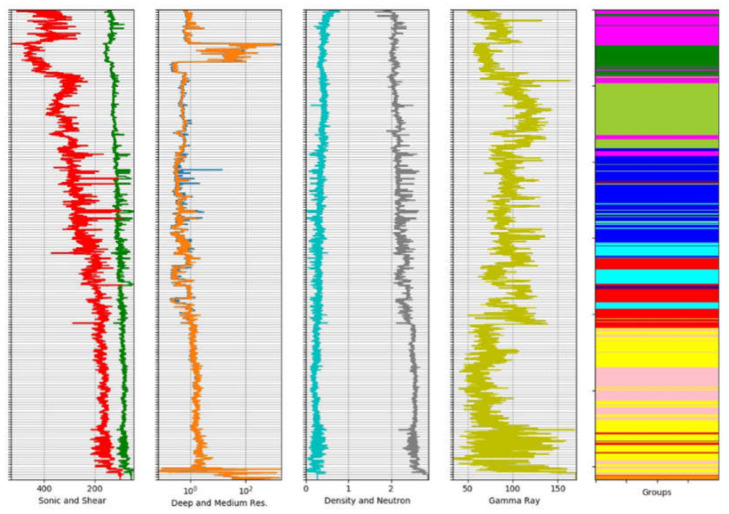
Groups obtained using k-Means (rightmost column) for 10 groups and a window of 6 m for well logs (from left to right, Sonic/Shear, Deep/Medium Resistivity, Density/Neutron and Gamma Ray). Clearly, the main lithologic groups (indicated by changes in well log patterns and values) were well defined by k-Means.

The results from the clustering process of well logs data were evaluated following the specialist’s criteria. The specialist also suggested the values for the window sizes utilized to divide the logs data, which were 3, 6, and 10 m. The windowing method was applied to seven well logs datasets: Deep Resistivity, Medium Resistivity, Sonic, Shear, Density, Neutron, and Gamma-Ray logs. Different configurations of k-Means (4, 6, 8, and 10 groups) were employed to obtain groups delimiting different lithologies present in the studied well. Figure 18 and Figure 19 provide a visualization of the clustering results of k-Means with 6 and 10 groups (rightmost column), respectively, both with a window of 6 m.

The examination of these figures indicates the effect in the output of the different number of groups. For 10 groups, the level of detailing regions with specific characteristics is higher, with great accuracy in their limits concerning the position of the data that generated the lithology. For example, the bottom region in Figure 19 presents very different characteristics, identified as the orange group in the figure, which is unique (not identified with other parts of the logs) and can be associated with volcanic rocks. In Figure 18, on the other hand, this area is not recognized as an independent group.

**Figure 20:**
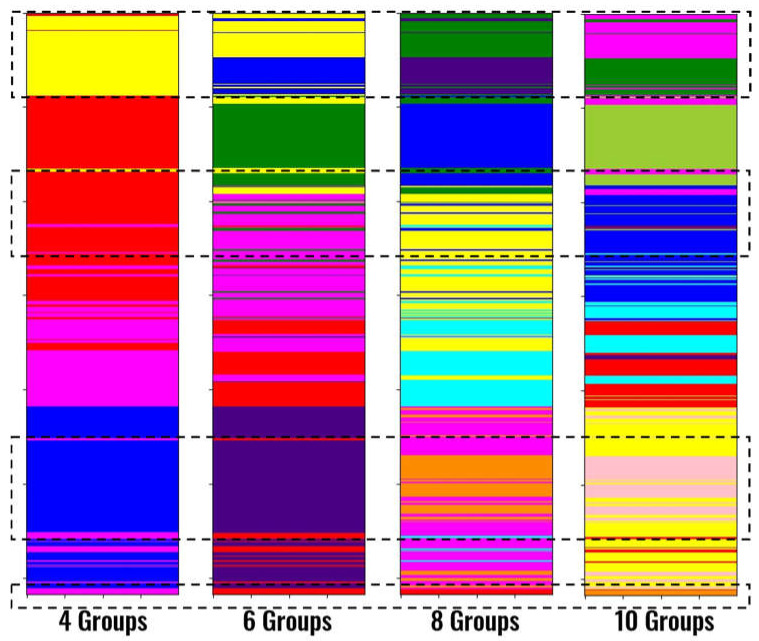
Results for different number of groups in k-Means for a window of 6 m.

This effect can be better observed in Figure 20, which displays the clustering results for different numbers of groups for a window size of 6 m. The regions surrounded by the dashed rectangles are some areas that are better detailed when the number of groups is higher than 4. The better identification of volcanic rocks is observed at the bottom.

**Figure 21:**
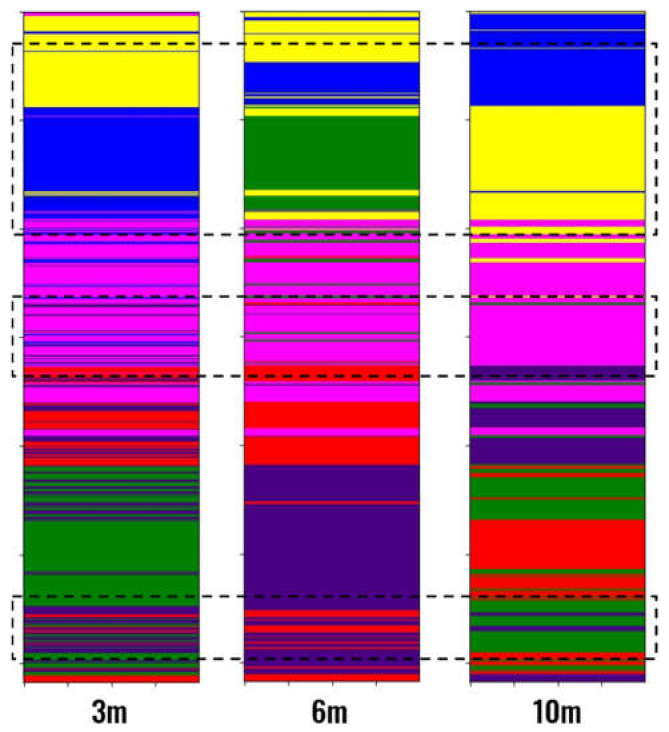
Results for different window sizes and k-Means with 6 groups.

When comparing the effects of the different window sizes evaluated (Figure 21), it can be seen that when the window size increases, the clustering result tends to be more regular, with larger groups and fewer partitions of a tiny size. With the decrease in the window size, scattered insignificant groups appear throughout all the results, which indicates that the clustering represents isolated portions of the well logs and not a meaningful part of the neighborhood. This behavior is highlighted in the areas inside the dashed rectangles. Therefore, it can be concluded that a window size of 3 m is not recommendable for this application.

### 3.3. Seismic and Well Logs Groups Association

**Figure 22:**
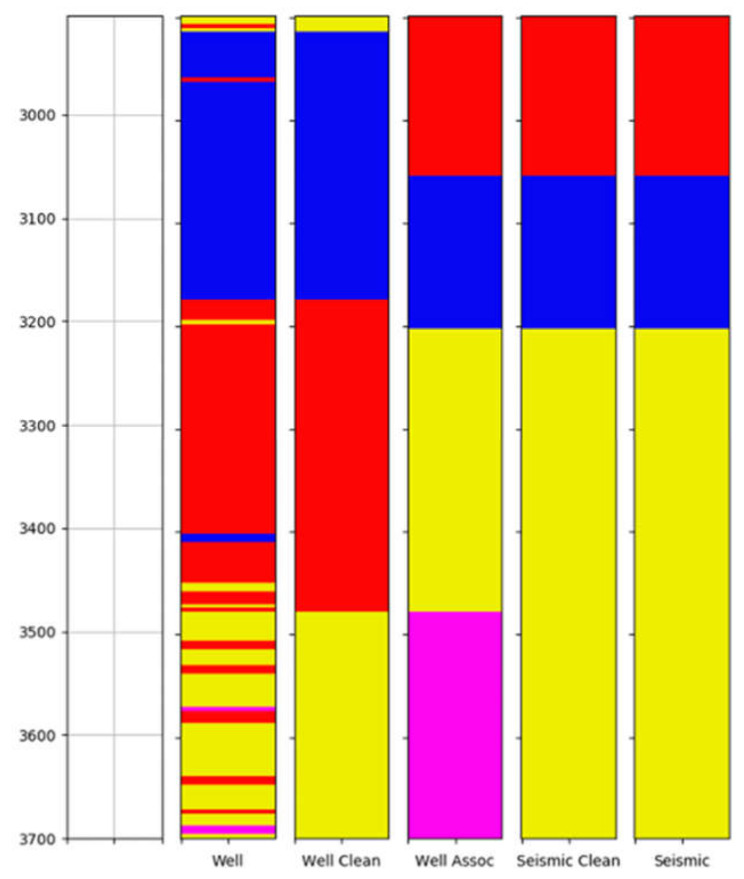
Association results.

**Figure 23:**
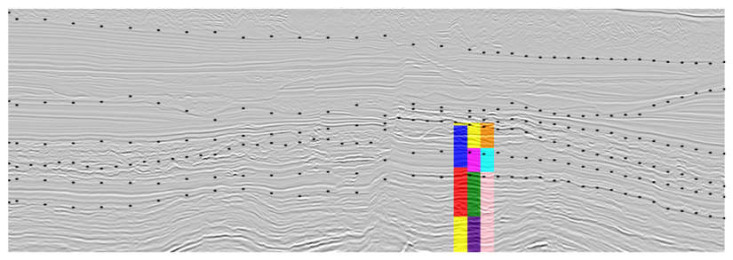
Example of association result, shown in color columns over seismic vertical slice. Black dots indicate main geological boundaries according to geophysicist interpretation on seismic data. Left column: clean well groups; Center column: association results; Right column: seismic groups. A very good fit is present for association results (central column) and seismic group (right column). Unfortunately, no well log information is available on the upper section of the seismic data, so only three seismic facies could be associated with the well log.

The last stage of this study is the association between the resultant seismic groups and well logs groups to produce an interpretation of the seismic data with aggregated lithologic information. This stage was validated by employing the most promising results evaluated by the specialist. The process involves several stages on the well logs data side in order to reduce its resolution and filter very small partitions, as described in Section 2.4. With these steps, it is clear that some little details are lost, and the groups represent the overall lithologies of this region. In general, the groups filtered from the well logs and those representing seismic facies were spatially correlated in all analyses, validating the unsupervised approaches. An example of the resultant association for the well clustering with a window of 6 m and six groups, and the seismic data as a composed image of Texture Contrast, Amplitude, Similarity Cross Average seismic attributes employing Watershed with the best subset of segment’s features (HOG, Neighborhood HOG, LBP, Neighborhood LBP) and eight groups is depicted in Figure 22 and Figure 23.

Figure 23 shows that the well size is almost half of the size of the seismic data. Therefore, the groups obtained from the upper half of the seismic image cannot be directly related to any information from the well logs groups, except for the case in which a group present in the well area is also present in the top half of the seismic image. In this specific case, the results from the three association methods are similar, which is repeated in most cases due to the most general distribution of groups (the well region is covered by a small number of groups in the seismic data). As a result, the present case relates the cyan and orange groups from the seismic clustering with the lithologic characteristics of the blue group in the well clustering, and the other group in the seismic clustering is related partly to the characteristics of the red one and partly to the properties of the yellow one belonging to the well columns.

## 4. Conclusions

This work presents the development of an unsupervised learning-based methodology to process seismic data. The produced groups were associated with the seismic facies obtained with lithologic information from the clustering of well logs data. The methodology compared two different pipelines that processed the seismic volume and the well logs separately. Two approaches were proposed to cluster the seismic data. The first one was based on clustering seismic attributes selected through the consensus of multiple feature selection algorithms. The second approach is based on interpreting the seismic volume as a sequence of images. In this case, the image is first segmented, generating segments of seismic data that are clustered to create the seismic interpretation. The well logs data were also clustered by employing windows of information and was associated with the groups representing seismic facies to include lithologic details in the final interpretation.

The methodology was developed in active collaboration with a specialist. As well as contributing with ideas related to the design of the processing pipeline and specificities of the data in both seismic and well logs domains, the specialist evaluated the results throughout the work. The unsupervised approaches produced groups with good relationships between variations of seismic facies and lithologies. When associated, the groups’ distributions also demonstrated a good spatial correspondence, which validated the proposed approaches.

The proposed methodology can assist the interpreter by producing a preliminary interpretation without any label, which significantly reduces the time required to finish the interpretation process. Additionally, the facies obtained are related to lithologic properties, creating a robust interpretation able to assist in the decision process of the hydrocarbon exploration activity.

The research presented in this paper can be expanded in several directions. Firstly, the proposed methodology should be evaluated in a different field with more wells to validate the synergy between seismic and well logs groups in multiple zones of the seismic volume. Secondly, an in-depth analysis of the relationship between the obtained seismic groups and lithological properties should be conducted, establishing a direct link between specific seismic attributes and lithologic properties that could be generalized to other fields. Additionally, the accuracy of the clustering results between close slices can be improved by establishing a postprocessing stage to correct small spatial dissimilarities between segments. Finally, novel deep learning-based unsupervised models can be evaluated on the seismic data, such as any variant of Autoencoders [61] or Generative Adversarial Networks (GANs) [62]. These deep learning models can also be exploited in combination with domain adaptation methods, utilising the knowledge obtained from different seismic data and extending the results—with improved accuracy—to data from new regions. Additionally, the texture information, which is demonstrated to be relevant in the clustering process, can be leveraged by its inclusion directly in the segmentation models, improving its final accuracy.

## Figures and Tables

**Table 1:** Parameters of clustering algorithms.

Algorithm.	Parameter	Value
k-Means	Groups	(4–8)
Kohonen	Groups	(4–8)
	Kohonen Map Size	(10 × 10–100 × 100)
	Grid Shape	Rectangular; Hexagonal
Agglomerative	Groups	(4–8)
	Distance Metric	Euclidean; Manhattan; Cosine; L1; L2
	Linkage	Ward; Complete; Average; Single

**Table 2:** Selection of subset of features to represent the segments. In bold are highlighted the features selected in each step.

First Step	Second Step	Third Step	Fourth Step
GLCM	GLCM	GLCM	Neighborhood (GLCM)
LBP	LBP	**LBP**	**Neighborhood (LBP)**
**HOG**	Neighborhood (All)	Shape	Neighborhood (Statistical)
Shape	**Neighborhood (HOG)**	Statistical (All)	Statistical (All)
Statistical	Statistical (All)	Neighborhood (All)	Shape
Neighborhood	Statistical (Histogram)	Neighborhood (GLCM)	GLCM
	Shape	Neighborhood (LBP)	
		Neighborhood (Statistical)	

## Data Availability

Publicly available datasets were analyzed in this study. This data can be found at http://geo.anp.gov.br/mapview searching for “Atlanta Field”.
